# Muscularization of the Mesenchymal Outlet Septum during Cardiac Development

**DOI:** 10.3390/jcdd7040051

**Published:** 2020-11-04

**Authors:** Maurice J. B. van den Hoff, Andy Wessels

**Affiliations:** 1 Department of Medical Biology, AmsterdamUMC, Location AMC, 1105AZ Amsterdam, The Netherlands; 2 Department of Regenerative Medicine and Cell Biology, Medical University of South Carolina, Charleston, SC 29425, USA; wesselsa@musc.com

**Keywords:** myocardialization, muscularization, differentiation, Wnt signaling, outflow tract

## Abstract

After the formation of the linear heart tube, it becomes divided into right and left components by the process of septation. Relatively late during this process, within the developing outflow tract, the initially mesenchymal outlet septum becomes muscularized as the result of myocardialization. Myocardialization is defined as the process in which existing cardiomyocytes migrate into flanking mesenchyme. Studies using genetically modified mice, as well as experimental approaches using in vitro models, demonstrate that Wnt and TGFβ signaling play an essential role in the regulation of myocardialization. They also show the significance of the interaction between cardiomyocytes, endocardial derived cells, neural crest cells, and the extracellular matrix. Interestingly, Wnt-mediated non-canonical planar cell polarity signaling was found to be a crucial regulator of myocardialization in the outlet septum and Wnt-mediated canonical β-catenin signaling is an essential regulator of the expansion of mesenchymal cells populating the outflow tract cushions.

## 1. Introduction

In the western world, approximately 3% of live-born children have a congenital abnormality. Of these children, a third have a cardiac defect, and of these children, again, a third have an arterial pole malformation. Over the past few decades, significant progress has been made in the understanding of the formation of the arterial pole. Professor Anderson has contributed nearly 200 publications to this effort. In this review, we will focus on one specific aspect of arterial pole formation, being the muscularization of the initially mesenchymal outlet septum. First, we will discuss the formation of the arterial pole to set the stage and then focus on myocardialization (for reviews of heart development, see [1,2]).

**Figure 1:**
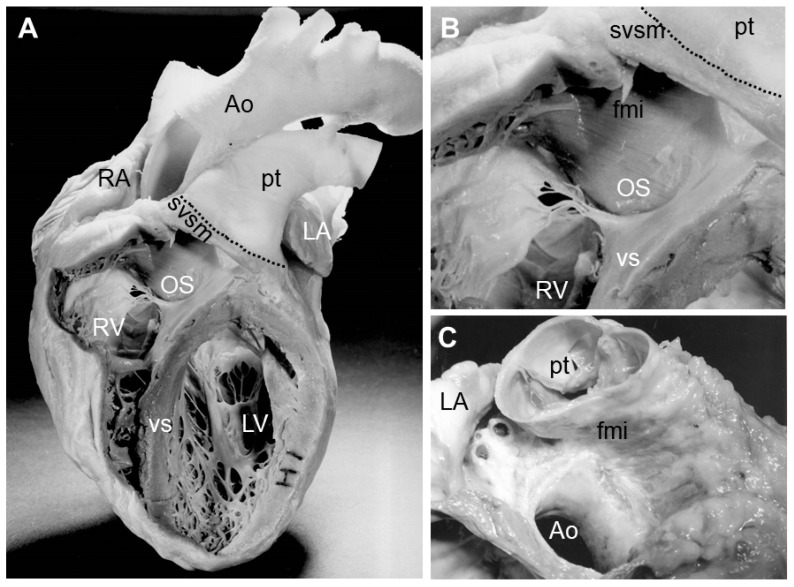
Images showing the distal myocardial border in the adult human heart. (**A**) shows an adult human heart in which windows were cut from the right and left ventricles to expose the interior structures. The dotted line indicates the distal myocardial border on the pulmonary trunk. (**B**) shows a detail of the windowed right ventricle focusing on the area of the outlet septum. (**C**) shows the relation of the large vessels leaving the heart. Note the difference in the level of the semilunar valves and the myocardial sleeve on the pulmonary trunk. The large vessels are cut at the distal myocardial border, showing the level of the myocardial border in relation to the semilunar valves. Abbreviations: Ao: aorta; fmi: freestanding muscular infundibulum; LA: left atrium; LV: left ventricle; OS: outlet septum; pt: pulmonary trunk; RA: right atrium; RV: right ventricle; svsm: semilunar valve supporting myocardium; vs: ventricular septum.

The arterial pole of the adult heart comprises (1) the intrapericardial portion of the aorta and pulmonary trunk, (2) the valves and the surrounding myocardium, and (3) the subvalvular ventricular outlets [3,4,5,6] (Figure 1). The formation of the arterial pole of the heart starts at the end of the fourth week of human development (Carnegie Stage (CS) 10), embryonic day (E) 8 in mice, and Hamburger and Hamilton stage (HH) 11 in chickens. At this stage, the outflow tract (OFT) can be discriminated as a smooth-walled, myocardial, tube-like structure in between the site of the forming ventricular trabeculae and the border of the pericardial cavity [7,8]. At the outer surface of the heart, the proximal side of the OFT is marked by the ventriculoarterial groove and the distal side by the pericardial reflection. Up to CS15 in human, E11 in mouse, and HH23 in chicken, the OFT elongates by the addition of cardiomyocytes at its distal end. This population of cardiomyocytes is derived from the so-called second heart field (SHF) resulting from de novo differentiation of mesodermal cells into cardiomyocytes [9,10,11,12,13]. During this process, the distal myocardial border of the OFT remains at the pericardial reflection and the pericardial cavity increases in size. At the same time, the cardiomyocytes at the proximal side of the OFT differentiate into ventricular cardiomyocytes, contributing to the right ventricle and interventricular septum [14,15]. Intriguingly, at the outer surface of the heart, the ventriculoarterial groove still marks the proximal site of the OFT, suggesting that the cells that form this groove are constantly changing. With the formation of the right ventricle, also the interventricular groove on the outer surface of the heart becomes apparent, marking the position of the forming interventricular septum. With development of the right ventricle, the distance between the ventriculoarterial and interventricular grooves increases in size. After CS15 (sixth week of human development, HH23 in chicken, and E11 in mouse), the OFT increases further in length due to the addition of non-myocytes to its distal border. As a consequence, the distal myocardial border of the heart is no longer found at the level of the pericardial reflection. This non-myocardial portion is referred to as the non-myocardial OFT and will become the intrapericardial portion of the aorta and pulmonary trunk. With further development, the non-myocardial OFT increases in length and the myocardial OFT becomes (relatively) shorter. Although most of the OFT myocardium differentiates into the right ventricular myocardium, a portion remains as OFT myocardium, supporting the semilunar valves and the smooth-walled subvalvular myocardium [14,16,17].

## 2. Septation of the Arterial Pole

The OFT comprises not only cardiomyocytes but also an inner layer of endocardial cells. The myocardium and endocardium are separated by a layer of extracellular matrix (ECM), referred to as a cardiac jelly. Prior to the onset of the formation of the non-myocardial OFT, the cardiac jelly of the OFT forms two major spiraling cushions, the parietal and septal OFT cushions (also referred to as OFT ridges). The cushions swell and fuse midway, separating the left and right bloodstreams. The fusion of the cushions starts from the distal side and proceeds proximally, i.e., in the direction of the ventricles. During this phase of development, the non-myocardial OFT forms (as described above) and it is important to note that no cardiac jelly is found in between the endocardium and the wall of the non-myocardial OFT. The lumen of the non-myocardial OFT connects the OFT lumen with the lumen of the aortic sac and the pharyngeal arch arteries that are located outside the pericardial cavity. The pharyngeal arch artery system shows extensive remodeling, which we will not discuss as it is beyond the scope of this review. Of relevance for this review is, however, that the left fourth pharyngeal arch artery will contribute to the aorta, and the left and right sixth pharyngeal artery to the left and right pulmonary artery, respectively. From the dorsal wall of the aortic sac, in between the origins of the fourth and the sixth pharyngeal arch arteries, the roof bulges into the lumen of the aortic sac at CS16 to form the aorticopulmonary septum (APS). The APS then expands in the direction of the fused OFT cushions. With its lengthening and the eventual fusion of its tip with the distal border of the fused OFT cushions, the aortic and pulmonary bloodstreams are separated [4,5,18].

During subsequent development, the non-myocardial OFT is separated into the intrapericardial portion of the aorta and pulmonary trunk. As a consequence, the APS is no longer recognized as a septum, but materially contributes to the facing walls of the intrapericardial portion of the aorta and pulmonary trunk. The separation of the arterial pole continues in the direction of the ventricles. The septation of the distal portion of the OFT results in completing the myocardial cuffs that support the forming semilunar valves. At this stage of development, the coronary orifices are still embedded within the myocardium. With ongoing development, the distal myocardial border further decreases to a level below the coronary orifices, approximately halfway the semilunar cusps. The disappearance of this last section of the myocardium is not only due to differentiation of the OFT myocardium into the ventricular myocardium but also due to apoptosis [14,19,20]. The extent of this process is different in the aortic and pulmonary segment, which is probably responsible for the fact that the primordia of the valves and, eventually, the formed aortic and pulmonary valves are no longer found at the same level but rather tilted with respect to each other. As a consequence of these changes, the myocardial cuff of the pulmonary outlet facing the aorta is now recognized as the freestanding muscular infundibulum. Thus, this free-standing muscular infundibulum is simply a myocardial sleeve supporting the leaflets of the pulmonary valve. It is only below the level of the aortic semilunar valves and distally of the membranous septum that a muscular septum persists.

## 3. Myocardializaton of the Outlet Septum

**Figure 2:**
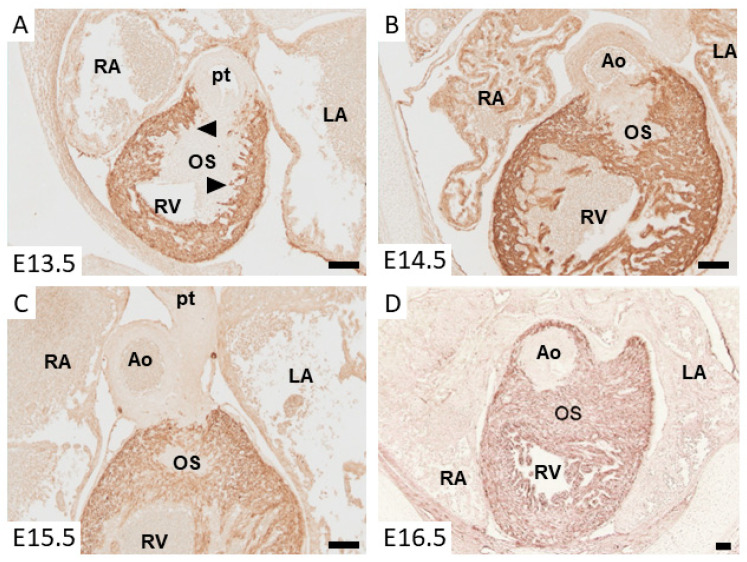
Micrographs of immunohistochemically stained sections of mouse embryos at the level of the outlet septum. The cardiomyocytes expressing ventricular myosin heavy chain are identified by their brown/red staining. (**A**) shows a section of the arterial pole at embryonic day (E) 13.5. Note the cardiomyocytes that protrude from the OFT myocardium into the mesenchyme of the outlet septum (arrow heads), being a hallmark of the onset of myocardialization. At E14.5 (**B**), the mesenchymal outlet septum is muscularized and becomes smaller. At E15.5 (**C**), only a small part of the mesenchymal outlet septum is found, and at E16.5 (**D**), the entire outlet septum is muscularized. The bar indicates a length of 100 µm. Abbreviations: Ao: aorta; LA: left atrium; OS: outlet septum; pt: pulmonary trunk; RA: right atrium; RV: right ventricle.

Prior to the onset of fusion of the OFT cushions, they become populated by mesenchymal cells. In the proximal part of the OFT, the cushions, the mesenchymal cells are mainly derived from the endocardium, as a result of endocardial to mesenchymal transition (endMT) [21]. In the distal as well as the middle part of the OFT, the cushions are primarily populated by mesenchymal cells that originate from the cardiac neural crest. These cardiac neural crest cells form a condensed pillar in both cushions located in the middle part of the OFT and become dispersed and intermingle with the endocardially-derived mesenchymal cells in the cushions of the proximal part of the OFT [16,22,23,24]. When the cushions in the proximal OFT are fused, the dispersed neural crest cells enter into apoptosis and the cardiomyocytes of the initially smooth-walled myocardium start to form protrusions into the flanking mesenchyme [16,20,25,26] (Figure 2). While invading the mesenchyme, they never lose their contact with the flanking OFT myocardium [16,27,28,29]. This process is referred to as myocardialization and continues until the entire outlet septum is myocardial. In the mouse, myocardialization takes place between E11 and E15 [30], in chicken between HH28 and HH38 [28], and in human between CS17 and CS23 [17].

## 4. Congenital Cardiac Abnormalities and Absence of Myocardializaton

Now that we have described the process of myocardialization, there are two (related) questions that need to be addressed: What would be the consequence of failure of myocardialization to take place? Are there congenital cardiac malformations that could be attributed to abnormal myocardialization of the proximal OFT?

If cardiac formation was normal and only myocardialization was absent, one would expect to find a fibrous continuity between the leaflets of the aortic and pulmonary semilunar valves, which are positioned at the same level, and a mesenchymal outlet septum in the formed heart. A congenital cardiac abnormality resembling these features is the doubly committed ventricular septal defect (VSD), the rarest form of VSD. In this situation, there is a fibrous continuity between the aortic and pulmonary semilunar valves and a VSD situated immediately below both the aortic and pulmonary semilunar valves, i.e., in the position of the outlet septum. In this situation, the position of the pulmonary and aortic outlets can be normal, or one or both can override the ventricular septum. In case the VSD is committed to either the aortic or the pulmonary outlet, the muscular freestanding infundibulum is formed. Another situation to be considered is the double outlet right ventricle (DORV). DORV is a very heterogenous group, which has in common the fact that both the aorta and pulmonary trunk emanate from the right ventricle. In addition, there is an obligatory communication between the left and right ventricle, of which a small portion has a doubly committed VSD. In case of a transposition of the great arteries, the connection of the aorta and pulmonary trunk is discordant with respect to the ventricles, but the muscular freestanding infundibulum and muscular outlet septum are formed. In case the OFT is not septated at all, as is the case in a common arterial trunk (CAT), it is evident that the freestanding muscular infundibulum and the muscular outlet septum are not formed. In CAT, absence of myocardialization is most probably secondary [3,31,32].

## 5. Mechanism of Cardiac Muscle Formation in the Outlet Septum: Muscularization vs. Differentiation

The mechanism by which the outlet septum changes from a mesenchymal into a myocardial structure is still contentious. Initially, we have described muscularization of the outlet septum as the result of migration of existing proximal OFT cardiomyocytes into the flanking mesenchyme, a process that we named “myocardialization” [28]. Migration was proposed based on the following in vivo and in vitro observations. (i) Prior to the onset of myocardialization in vivo, the myocardial wall flanking the cushion mesenchyme is smooth. The first signs of myocardialization are the appearance of protrusions from the proximal OFT myocardium into the flanking cushion mesenchyme. With ongoing development, the cardiomyocytes that intermingle with the cushion mesenchyme remain slender and show projections. (ii) Culturing chicken or mouse OFT explants (of stages that do show myocardialization in vivo) on a thick 3D collagen lattice showed spontaneous 3D network formation of cardiomyocytes below and surrounding the explant in the collagen lattice. OFT explants prepared of stages prior to myocardialization in vivo, distal OFT explants, or ventricular explants of any developmental stage did not show network formation when tested in this assay. When OFT explants prepared of stages prior to myocardialization in vivo were cultured in medium conditioned by OFT explants that do form a myocardial network in the collagen, they can be induced to do so. This conditioned medium was not able to induce myocardialization in ventricular or distal OFT explants. Moreover, medium conditioned by ventricular explants did not induce myocardial network formation of young OFTs [28,30]. These findings support the hypothesis that the myocardialization-inducing substance is a factor secreted by cells of the OFT at stages when myocardialization is observed in vivo. 

In an attempt to further characterize myocardialization, proximal OFT cushion mesenchyme was cultured in the myocardialization assay. Cushion mesenchyme did not form cardiomyocytes or networks. However, when proximal OFT cushion mesenchyme was cultured in medium conditioned by OFT explants that do form myocardial networks in vivo, cardiomyocytes were formed. This effect was found to be specific for the proximal OFT cushion mesenchyme, because explants of the non-myocardial OFT were not able to do so [30]. Both in vitro and in vivo, the expression of myosin heavy chain in the cells forming the myocardial network is proceeded by alpha smooth muscle actin [17,33,34] or calponin [35] expression, being suggestive of a differentiation process. It should, however, be noted that smooth muscle actin expression has also been used as a marker for migration [36]. Taken together, these findings suggest that muscularization of the outlet septum might not only be the result of myocardialization but that differentiation of the proximal OFT cushion mesenchyme may also contribute. 

If cardiomyocytes are indeed newly formed as a result of differentiation, one needs to consider the origin of the differentiating cells. The mesenchyme of the outlet septum is derived from the endocardium, cardiac neural crest [23,24], and the epicardium (unpublished observations). The epicardially derived mesenchyme seems to be an unlikely cell source for myocardial differentiation in the context of myocardialization of the OFT as epicardially derived cells arrive after the onset of muscularization in the outlet septum. Neural crest-derived mesenchyme can also be excluded because these cells undergo apoptosis prior to the onset of the muscularization [16,25,37]. Furthermore, the non-myocardial OFT, which is largely derived from cardiac neural crest cells, is not able to form myocardial networks in the in vitro 3D collagen assay [28]. Taken together, this points to the endocardially derived mesenchyme as a possible source of newly differentiated cardiomyocytes. This idea is supported by the observations that (i) the endocardial derived cells are abundantly present at the proper time and location and (ii) that endocardial cells are derived from the cardiogenic mesoderm [15,38,39,40]. Importantly, however, genetically tracing the endocardial cell lineage using the Tie2-Cre; ROSA26 model does not identify cardiomyocytes that are of endocardial origin. The latter finding suggests that if any muscularizing cells in the outlet septum have formed as a result of a process of myocardial differentiation, it is not as a result of differentiation of any cell type in the OFT that we know of to date.

## 6. Molecular Regulation of Myocardium Formation in the Outlet Septum

The in vitro experiments with conditioned medium discussed above suggest that muscularization of the outlet septum might be induced by a secreted soluble factor. Efforts to identify the nature of this factor from conditioned medium have not yet been successful. In a candidate gene approach, a large set of growth factors was tested for their ability to induce myocardial network formation in early OFT explants. These experiments showed that, when tested individually, activin A, angiotensin II, BMP2, BMP4, cardiotrophin, endothelin-1, -2, -3, FGF2, IGF-II, neurotrophin-3, osteopontin, or TGFβ1 were not able to induce myocardial network formation. However, TGFβ2 and TGFβ3 were, in a concentration-dependent manner, able to induce the formation of myocardial networks [41]. In studies analyzing mesenchyme formation in atrioventricular explants, TGFβ2 or TGFβ3 have been identified as important regulators of endMT and to regulate the migration of the mesenchymal cells into the acellular cushions (for review, see [42]). In vitro analyses also uncovered a role for the TGFβ receptors in the regulation of endEMT (for review, see [43]). A difference in the spatiotemporal expression pattern of the TGFβ receptors might underlie the difference in the effect of TGFβ2 and TGFβ3 in myocardialization. However, this idea has, to the best of our knowledge, not been pursued. In mice, deletion of TGFβ2 [44,45] was found to affect the development of the OFT to different degrees. In several of the TGFβ2 knockout embryos, the cushions in the proximal OFT were underdeveloped, had not fused, and were not muscularized. When OFT explants of wildtype and TGFβ2 knockout mice were studied in the 3D collagen myocardialization assay, no difference in myocardial network formation was found [46]. These results suggest that aberrant myocardialization is not a direct effect of the absence of TGFβ2, but rather a secondary effect related to mesenchyme formation in the cushions. Although, in the first report describing TGFβ3 knockout mice, cardiac defects were not reported [47], a recent study reported a small fraction of TGFβ3 knockout embryos in which the OFT cushions were not fused or myocardialized [48].

The abundance of mesenchymal cells in the OFT cushions seems to be of relevance in the regulation of myocardialization, because the level of dysregulation of endMT in the TGFβ2 and TGFβ3 knockout models is different. Another example of a mouse model with abnormal cushion development and failure of muscularization is the neurofibromin 1 (Nf 1) knockout mouse in which the OFT cushions are hyperplastic and are not muscularized [49]. Interestingly, in the friend-of-GATA 2 (Fog2) knockout mouse, the proximal parts of the OFT cushions are hyperplastic and muscularization does take place [50]. The major difference between these latter two mouse models seems to be the extent of apoptosis of mesenchyme in the proximal part of the OFT. During normal development, the dispersed neural crest cells in the proximal part of the OFT cushions enter into apoptosis prior to myocardialization. In the Fog2 knockout mouse, the level of apoptosis appears normal, perhaps even elevated, whereas in the Nf1 knockout mouse, the level is significantly decreased. Further analysis of the Nf1 knockout mouse showed that the OFT cushions are enlarged due to expansion of the endocardially derived cell population, which seems to hamper the invasion of neural crest cells [49]. Thus, the absence of apoptosis in the Nf1 knockout mice is due to the absence of the neural crest cells rather than a dysregulation of apoptosis. The presence of the dispersed neural crest cells and their subsequent apoptosis seems to be an important aspect in the regulation of myocardialization.

Deletion of semaphorin-3c (Sema3c) from the neural crest cells or absence of its receptor neuropilin-1 (Nrp1) interferes with the invasion of the neural crest cells. In this model, the mesenchymal cells in the cushions in the proximal part of the OFT are disorganized and myocardialzation is absent. Interestingly, the cardiac muscle cells in these mutant mice show the phenotypic characteristics of myocardialization in the distal portion of the OFT [51]. Ectopic myocardialization in the distal OFT was also found in the Trisomy 16 mouse [52]. In this study, ectopic myocardialization was suggested to be associated with the location of the invading cardiac neural crest cells. In connexin 43 (Cx43) knockout mice, which display OFT and right ventricular abnormalities, a reduction in the number of invading cardiac neural crest cells was observed [53]. In a detailed analysis, myocardialization was found to be affected, as the cardiomyocytes of the proximal part of the OFT were found to form relatively late and only a few projections into the cushion mesenchyme were observed. Furthermore, the myofibrillar structure in these cardiomyocytes was disorganized [54]. A similar defect was observed in the loop-tail mouse. In a series of manuscripts by the Henderson group, it was established that in these mice, Vangl2, expressed in the cardiomyocytes, was mutated. Vangl2 is a component of the Wnt-planar cell polarity (Wnt/PCP) signaling pathway [55]. Downstream of Vangl2, RhoA and Rock1 were found to be required for the polarization and movement of the cardiomyocytes into the OFT cushions [56,57,58]. Disrupting the interaction of Prickle1 with Vangl2 was found to lead to similar abnormalities [59]. Interestingly, Cx43 expression also seems to be linked to the Wnt/PCP pathway through Rock1, which is downregulated in Cx43 KO mice [60].

It has not yet been established which Wnt ligand activates the Vangl2-mediated signaling pathway. Based on the analysis of genetically modified mice, Wnt5a and Wnt11 are candidates. In Wnt11 knockout mice, transposition of the great arteries (TGA) is observed. An in-depth analysis revealed that the freestanding muscular infundibulum and muscular outlet septum were not formed [61,62] due to altered polarity of the cardiomyocytes of the proximal part of the OFT [62]. Wnt11 expression is regulated through the cooperative action of Pitx2 and β-catenin on its proximal promoter in cardiomyocytes. Wnt11, in turn, activated TGFβ2 through JNK and ATF2 [62], pointing to an indirect effect on endEMT. Wnt5a knockout mice show a large array of abnormalities, including common arterial trunk (CAT). Expression of Wnt5A in the mesodermal cells of the pharyngeal arches and in the myocardium of the OFT [63] was found to regulate the migration of cardiac neural crest cell into the OFT. The myocardial expression of Ror 1 and Ror 2 receptors [64], which propagate Wnt5a mediated PCP signaling [65], is suggestive of a role in the regulation of myocardialization. This idea is underscored by the observations that Ror1/Ror2 double knockout mice display TGA and a membranous VSD, whereas Ror1 knockout mice have a normal heart and Ror2 knockout mice have a membranous VSD [66,67]. 

Wnt5a was also found to activate Wnt/β-catenin-mediated signaling through Frizzled-4 [68]. Interestingly, a mutation screen identified Frizzled-4 in a mouse line showing a large VSD in the region of the outlet septum. Further analysis of this mouse line showed that the VSD was due to aberrant migration of the cardiac neural crest cells into the OFT [69]. In Dishevelled-2 (Dvl2) knockout mice, perturbed neural crest migration into the OFT was observed, resulting in CAT and DORV [70]. This observation should be interpretated with caution as in mouse and human, three homologous Dvl genes are found that are expressed abundantly. Knockout analysis has shown that they have complementary and unique functions. Dvl was found to activate both Wnt/β-catenin and Wnt/PCP signaling pathways. The specificity of the signaling is regulated through different domains in the Dvl protein and interacting partners and as such affect different biological effects [55].

Conditional removal of focal adhesion kinase (FAK), which is also a component of Wnt/β-catenin-mediated signaling, from the cardiomyocytes using Nkx2.5-Cre results in an overriding aorta or DORV. In this model, cardiomyocytes do not invade the proximal OFT cushions, and in in vitro assays, it was observed that cardiomyocytes isolated from homozygous FAK knockout mice were not able to migrate. Further analysis of the underlaying signaling pathways identified FAK-dependent CAS (Crk-associated substrate) activation as an essential regulator of polarized myocyte movement [71].

The involvement of FAK-dependent CAS in the regulation of myocardialization links Wnt/β-catenin-mediated signaling with integrin-mediated signaling. Integrins are the main receptors transferring extracellular matrix (ECM) information into the cells. Thus far, altered integrin expression has not been considered in the context of myocardialization. The ECM is not only of importance for the migration of cardiomyocytes into the OFT cushions, but also for the population of the cushions by mesenchymal cells. The mesenchymal cells of the proximal OFT cushions might not only directly but also indirectly affect myocardialization through ECM modulation. In this respect, it is interesting to note that both in vitro and in vivo analyses showed that low levels of fibronectin are required for intermingling of cardiomyocytes and mesenchymal cells, whereas high levels of fibronectin inhibit their intermingling [72]. Moreover, altering the relative abundance of splice variants of the chondroitin sulfate proteoglycan versican was observed to result in underdeveloped OFT cushions and absence of myocardialization [73]. These findings point to a role of the ECM in supporting or regulating myocardialization and warrant further analysis.

Taken together, the regulation of muscularization of the cushions in the proximal OFT is a complex process in which Wnt and TGFβ signaling play essential roles and where the interaction between cardiomyocytes, endocardial derived cells, neural crest cells, and the extracellular matrix seems to be essential.

## 7. Muscularization of the Dorsal Mesenchymal Protrusion

Although this review primarily deals with the process of muscularization of the outflow tract, it seems appropriate to also briefly pay attention to one other part of the developing heart where a mesenchymal structure eventually becomes muscularized. This structure is the dorsal mesenchymal protrusion (DMP), which plays a crucial role in atrioventricular septation together with two other mesenchymal structures: the AV cushions and the mesenchymal cap on the leading edge of the primary atrial septum (for review, see [74]). The DMP, which is sometimes also referred to as the spina vestibula or the vestibular spine, is a second heart field-derived structure [75,76]. In the early stages of atrioventricular septation (in the mouse between E9.5 and E10.5), the DMP protrudes into the common atrium, using the dorsal mesocardium as its portal of entry. It will eventually fuse with the other mesenchymal component and, as part of the AV mesenchymal complex, sits on top of the fused AV cushions, forming the base of the atrial septal complex. It is not until E13 in the mouse that the DMP becomes muscularized and becomes the inferior muscular rim of the atrial septum. Muscularization of the DMP was initially considered to take place by myocardialization [29,30,77]. However, more recent work from us and others shows that the DMP becomes muscularized as a result of a mesenchymal-to-myocardial differentiation of SHF-derived mesenchyme [75,78,79,80,81,82]. 

## 8. Concluding Remarks

During cardiac septation, a mesenchymal outlet septum is formed in the outflow tract by the fusion of the OFT cushions. During subsequent development, this mesenchymal outlet septum becomes muscularized by the process of myocardialization, which is defined as the migration of existing cardiomyocytes into flanking mesenchyme. Detailed analyses of myocardialization using genetically modified mice and in vitro assays suggest that the relative contribution of each cell population and the interaction between endocardially derived mesenchymal cells, cardiac neural crest-derived mesenchymal cells, and cardiomyocytes in the outlet septum is essential for the correct orchestration of the transition of the cardiomyocytes into a migratory phenotype and their migration into the adjacent cushion mesenchyme. The migration of cardiomyocytes into the mesenchyme is mediated by the Wnt non-canonical planar cell polarity signaling and the expansion of the mesenchymal cells in the OFT cushions by Wnt-mediated canonical β-catenin signaling. In addition, the TGFβ signaling pathway seems to be important in the regulation of myocardialization by affecting the mensenchymal cell population of the cushions. 

While the importance of muscularization/myocardializion for proper heart development is evident, it is tempting to speculate about the importance of this mechanism in a broader context. A myocardial infarction results in loss of cardiomyocytes and subsequent replacement by fibrous scar tissue. The limited inherent regenerative capacity of the adult cardiomyocytes prevents the repopulation of the scar (for review, see [83]). As a therapeutic approach to repopulating the fibrous scar and, hence, preserving cardiac function, insight into the mechanisms that regulate myocardialization in the embryonic heart may also be of relevance in designing strategies to induce healthy cardiomyocytes from the border zone of the infarct to migrate into the scar and improve cardiac function.
